# Disability‐Adjusted Life Years (DALYs) due to Breast, Cervical, Colorectal and Oral Cancers in Taiwan Regions

**DOI:** 10.1002/cam4.70592

**Published:** 2025-01-07

**Authors:** Cheng‐Chieh Hsieh, Si‐Yu Chen, Chun‐Hui Lin, Szu‐Chieh Chen, Chung‐Min Liao

**Affiliations:** ^1^ Department of Public Health Chung Shan Medical University Taichung Taiwan; ^2^ Department of Bioenvironmental Systems Engineering National Taiwan University Taipei Taiwan; ^3^ Department of Surgery, Division of General Surgery Feng Yuan Hospital, Ministry of Health and Welfare Taichung Taiwan; ^4^ Department of Family and Community Medicine Chung Shan Medical University Hospital Taichung Taiwan

**Keywords:** cancer burden, county scale analysis, disability‐adjusted life years (DALYs), Taiwan regions

## Abstract

**Background:**

Cancer is a leading cause of death globally, with significant variations in incidence and mortality rates among different cancer types and regions. In Taiwan, breast cancer (BC), cervical cancer (CxCa), colorectal cancer (CRC), and oral cancer (OC) are prevalent and have prompted government‐led screening programs to mitigate their impact. This study aims to assess the burden of these cancers at the county scale using disability‐adjusted life years (DALYs) as a metric, focusing on the years 2010, 2015, 2018, 2019, and 2020.

**Methods:**

Data on cancer incidence, mortality, disability weights, and treatment outcomes were sourced from the Taiwan HPA, Ministry of Health and Welfare, and Taiwan Cancer Registry. Years of life lost (YLLs) and years lived with disability (YLDs) were calculated for each cancer, considering age, stage, and treatment. The correlation between cancer screening rates and disease burden also conducted.

**Results:**

The analysis highlights significant trends in cancer mortality, incidence, and disease burden in Taiwan from 2010 to 2020. BC and CRC showed rising ASMR and DALYs rates, while CxCa experienced consistent declines. OC had a fluctuating pattern, particularly in eastern regions. YLLs contributed significantly to DALYs for all cancers, emphasizing premature mortality's role in the disease burden. Screening rates, particularly for BC and CxCa, correlated with changes in burden, with BC rates increasing and CxCa decreasing, reflecting the impact of preventive measures on cancer outcomes.

**Conclusions:**

The findings underscore the importance of targeted interventions and evidence‐informed resource allocation to address regional differences in cancer burden in Taiwan.

## 1 Introduction

Cancer remains a major global health challenge, with increasing incidence and mortality rates across various regions, including significant differences in cancers known as breast cancer (BC), cervical cancer (CxCa), colorectal cancer (CRC), and oral cancers (OC) [1, 2]. A recent analysis from the Global Burden of Disease Study 2021 provided important data on health metrics such as prevalence, incidence, years lived with disability (YLDs), years of life lost (YLLs), disability‐adjusted life years (DALYs), and healthy life expectancy (HALE) [3]. DALYs are particularly useful in estimating the impact of disease‐related disability or premature mortality on population's health [4].

While much research has focused on cancer burden at the global, regional, and national scales [5, 6], there are few studies that delve into the burden at the county scale [7, 8, 9]. In Europe, disease burden studies are also conducted at the single‐country (59%) or multi‐country (41%) scales [10]. Studies in Brazil, for example, has shown county‐specific significant differences in cancer burden [7]. Similarly, Yang et al. (2021) [8] analyzed the disease burden among 33 administrate areas and found that DALY rates for OC varied threefold among the counties with the lowest and highest burdens in China. In addition, in the US [9], disparities in screening rates were observed among counties with different levels of social vulnerability.

In Taiwan, cancer has become a growing health concern due to a rapidly aging population and lifestyle changes [11]. Since 1995, Taiwan's national health insurance has included free screenings for cervical and colorectal cancers, with expanded outreach for breast and oral cancer by 1999. These national screening programs not only have offered tests but also established a screening information system and a cancer registry to ensure quality monitoring [12, 13, 14].

Currently, the Health Promotion Administration (HPA) in Taiwan provides subsidized cancer screenings for several cancers, targeting specific age groups and high‐risk populations. Women aged 45–69, as well as women aged 40–44 with a close relative who has had BC, can receive a mammogram every 2 years. For CxCa, women aged 30 and above are eligible for a Pap smear every 3 years. People aged 30 and above who chew betel nut or smoke, as well as indigenous people aged 18 and above with the same habits, can receive an oral mucosal examination every 2 years to screen for OC. Additionally, individuals aged 50–74 have had access to government‐subsidized fecal occult blood tests every 2 years over the past decade to screen for CRC. These four screenings, recommended by the WHO, are part of the National Cancer Screening Program [15].

The county‐specific variation in disease burden may be linked to the availability of screening resources and different risk factors across areas. Furthermore, few studies have scientifically and objectively assessed major cancer burdens and their characteristics in more detail by region on the county scale. In addition, focusing on the county scale case study has empirical values, providing a reference for the basic research and implementation of cancer burden assessment in other counties at the global scale. Therefore, this study aims (1) to analyze the temporal trends in age‐standardized mortality rates (ASMR) and age‐standardized incidence rates (ASIR) of BC, CxCa, OC, and CRC in Taiwan from 2010 to 2020, using the DALYs approach to illustrate short‐, medium‐, and long‐term trends; (2) to conduct a county‐level spatial analysis of the cancer burden in Taiwan; (3) to evaluate the contribution of YLL to the overall cancer burden (DALYs) in Taiwan; and (4) to assess the impact of screening programs on the age‐standardized DALYs rates for these cancers, investigating the correlation between screening rates and changes in the overall disease burden across different regions of Taiwan.

## 2 Materials and Methods

### 2.1 Study Data Sources

**FIGURE 1:**
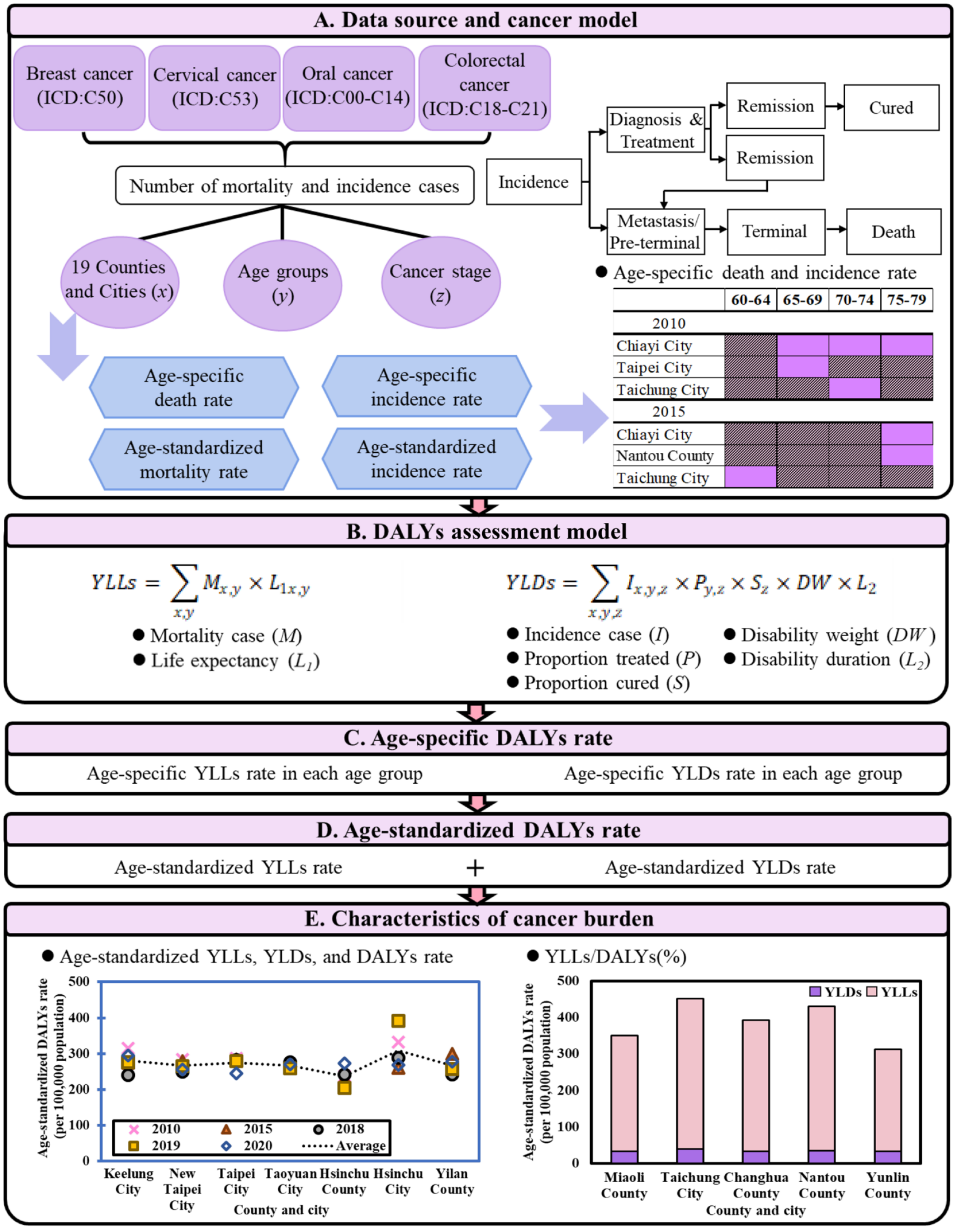
Schematic showing the overall comprehensive framework of the study: (A) data source and cancer model, (B) DALYs assessment model, (C) age‐specific DALYs rate, (D) age‐standardized DALYs rate, and (E) characteristics of cancer burden.

Figure 1 shows the overall comprehensive framework of the study algorithm. We used the statistical data and related epidemiological parameters: Proportion treated (*P*), proportion cured (*S*), disability weight (DW), and disability duration (*L*) for estimating the cancer burden due to premature death or disability caused by four cancers in 2010, 2015, 2018, 2019, and 2020 years. This analysis includes long‐term changes over a decade, medium‐term variations at 5‐year intervals, and short‐term trends immediately preceding 2020.

The incidence of cancer cases across age groups was sourced from the Taiwan HPA (2023) [16]. We focused on the four cancer sites based on the cancer screening policy in Taiwan nowadays, including OC (ICD‐10: C00‐C14), CRC (ICD‐10: C18‐C21), BC (ICD‐10: C50), and CxCa (ICD‐10: C53).

The cancer‐specific deaths by age groups from 2010 to 2020 in Taiwan were extracted from the Department of Statistics, Ministry of Health and Welfare (2023) [17]. The cases of cancer incidence and mortality were divided into age groups for different types of cancer: 0–39 years, 40–59 years, and 60+ years for OC; 0–49 years, 50–69 years, and 70+ years for CRC; 0–34 years, 35–49 years, 50–69 years, and 70+ years for BC; and 0–29 years, 30–49 years, 50–69 years, and 70+ years for CxCa. All datasets were classified into five cancer states (stages 0, I, II, III, and IV) and disease staging followed the 8th edition of the American Joint Committee on Cancer (AJCC_8th).

### 2.2 Burden Estimation

DALYs calculation in each confirmed case of OC, CRC, BC and CxCa was estimated by using YLLs and YLDs that are calculated separately before being merged into a unified summary metric as,

(1) DALYs=YLLs+YLDs

where YLLs were computed by multiplying the number of deaths specific to cancer at a particular age by the standard life expectancy for that age group as,

(2) $$\text{YLLs}=\sum_{x,y}M_{x,y}\times L_{1x,y}$$

where *M*_*x,y*_ indicates the number of deaths attributed to cancer within various countries and ages. The age categories (*y*) were collected according to surveillance protocols [16]. *L*_1*x,y*_ indicates the YLLs due to premature death within various counties and ages. The average life expectancy was adopted from statistical data from the Taiwan Ministry of the Interior [18].

On the other hand, YLDs were calculated by multiplying the number of new cases by the average duration of disability and the disability weights corresponding to the stages of the disease,

(3) $$\text{YLDs}=\sum_{x,y,z}I_{x,y,z}\times P_{y,z}\times S_{z}\times \mathrm{DW}\times L_{2}$$

where *I*_x,y,z_ indicate the country‐specific (*x*) incidence case at different age groups (*y*) and cancer stages (*z*); *P*_*y,z*_ represents the proportion treated by different age/cancer stage‐specific (y, *z*); *S*_*z*_ indicate the cancer stage‐specific (*z*) proportion cured; *L*_2_ represents the cancer phase‐specific disability duration and *DW* represent the cancer phase‐specific disability weight. In our study, we selected disability weights (*DW*s) for BC, CRC, OC, and CxCa from GBD 2019 Adolescent and Young Adult Cancer Collaborators (2022) [19]. These source was chosen because it provides a comprehensive and standardized set of disability weights based on global surveys and expert consensus. These weights were derived from extensive data collected through household interviews and online surveys conducted across multiple countries, ensuring that the estimates account for diverse linguistic, cultural, and socioeconomic contexts [20]. Although the GBD‐derived DW may not perfectly reflect the specific conditions in Taiwan due to the lack of localized data, they are widely recognized and applied in international health research.

### 2.3 Three‐Stage Natural History of Cancer

A three‐stage natural history of cancer is a conceptual framework used to understand the progression of cancer from earliest stages to advanced disease (Figure S1) [21]. The model outlines three possible pathways for newly diagnosed cancer cases. Briefly, individuals who received treatment (*P*) and were subsequently cured (*S*) of cancer experienced periods of disability during both the primary diagnosis and therapy phase (*L*_D_) and remission (*L*_R1_). Intensive follow‐up during remission aimed to detect any signs of recurrence or dissemination. On the other hand, those who succumbed to cancer after treatment (1‐*S*) faced disability in various phases: primary diagnosis and therapy (*L*_D_), remission (*L*_R2_), pre‐terminal (*L*_M_), and terminal (*L*_T_). Lastly, individuals who didn't undergo treatment (1‐*P*) encountered disability periods during the pre‐terminal (*L*_M_) and terminal (*L*_T_) phases.

To estimate the disease stage‐YLDs, this study combined Equation (3) with a three‐stage nature history of cancer, summing the burden for each cancer phase in different countries. Table S1 presents cancer‐specific disease weights (*D*_*D*_, *D*_*R*_, *D*_*M*_, and *D*_*T*_) and disease durations (*L*_*D*_, *L*_*R1*_, *L*_*R2*_, *L*_*M*_, and *L*_*T*_) in different cancer phases for four cancers. The mean and 95% UI of disability weights for each phase of the natural history of cancer were derived from GBD's study [19, 22, 23, 24].

### 2.4 Proportion Treated

The proportion treated (*P*) is determined by calculating the percentage of patients who underwent surgery, chemotherapy, radiotherapy, or a combination of these treatments. Table S2 shows the average proportion treated (*P*) from 2010 to 2020 at different cancer stages and age groups. For OC, the average *P* for each stage ranges from 95% to 99%. *P* decreased from 96% to 93% in the older age group (60+ years) at stage IV. Similarly, for the other three cancers, *p* values remain consistently high, exceeding 90%, except for the elderly population at severe cancer stage IV. For instance, in CRC, *P* drops from 93% (0–49 years) to 76% (70+ years), and in BC, it decreases from 82% (0–34 years) to 68% (70+ years), both at stage IV. In this study, we adopted data from the population‐based Taiwan Cancer Registry to calculate *p* for four selected cancers. Multiple combinations of treatments were documented, and we excluded instances related to “palliative care” in cancer, “untreated registry”, and the number of “Other treatment registry”.

### 2.5 Proportion Cured and Life Expectancy

The proportion cured (*S*) was obtained from a 5‐year survival report published by the Taiwan Health and Welfare Report [25]. Table S3 lists the proportion cured (*S*, %) in the period 2010–2020 at different cancer stages (stage 0, I, II, III, and IV) and cancer sites. In Taiwan, the average *S* for OC ranges from 78.0% to 35.9% across stages 0 to IV. BC and CxCa exhibit the highest *S* at stage 0, with an average of 99.0% and 98.1%, respectively. Conversely, CRC and CxCa have the lowest *S* at stage IV, with averages of 12.9% and 21.4%. For life expectancy calculations, we used a life table with variable life expectancy based on birth year available at https://www.moi.gov.tw/cl.aspx?n=3053 [18]. OC and CRC use all population life tables, while BC and CxCa specifically use female population life tables.

### 2.6 Statistical Methods

This study considered the differences in population size and age structure among administrative districts over time when comparing the burden. The metric of age‐specific death rate (ADR), age‐specific incidence rate (AIR), ASMR, age‐standardized incidence rate (ASIR), and age‐standardized DALYs rate per 100,000 population for four cancers were calculated. We used the direct standardized method to be consistent in population structure.

Firstly, the age‐specific rate was estimated, and the age‐specific burden of the year was divided by the mid‐year population and then multiplied by 100,000 population to give the burden per 100,000 population per year. The result will show the disease burden in different age groups as,

(4) $$\mathrm{Age}-\text{specific rate}=\frac{M_{y}}{N_{y}}\times 100,000$$

where *M*_*y*_ represents the number of incidence cases, deaths, or DALYs at different age groups (*y*) of the year; *N*_*y*_ indicates the mid‐year population of that year in different age groups (*y*). The mid‐year population was obtained from population by age for counties and cities from the Taiwan Ministry of the Interior [26].

Next, the age‐standardized rate was calculated as the age‐specific rate multiplied by that age group standard population percentage, and the sum of all the age group results in that the result will have the same age structure,

(5) $$\mathrm{Age}-\text{standardized rate}=\sum_{y}\mathrm{Age}-{\text{specific rate}}_{y}\times W_{y}$$

where *W*_*y*_ represents the percentage of each age group in the standard population in that the population used the world standard population with the unit of per 100,000 population per year [27].

We also performed a correlation analysis between DALYs and screening rates for four specific cancers to assess the potential association between the extent of cancer screening and the disease burden. Pearson's correlation coefficient was used for evaluating the strength and direction of the linear relationship between two continuous variables. This approach enabled us to determine whether higher screening rates correlate with a reduced disease burden, as indicated by lower DALYs. Besides, our study complied with the standardized reporting guidelines for burden of disease studies [28]. The Standardize reporting of burden of disease studies (STROBOD) checklist were listed in Table S4.

## 3 Results

### 3.1 County‐Specific Cancer Mortality and Incidence Rate

**FIGURE 2:**
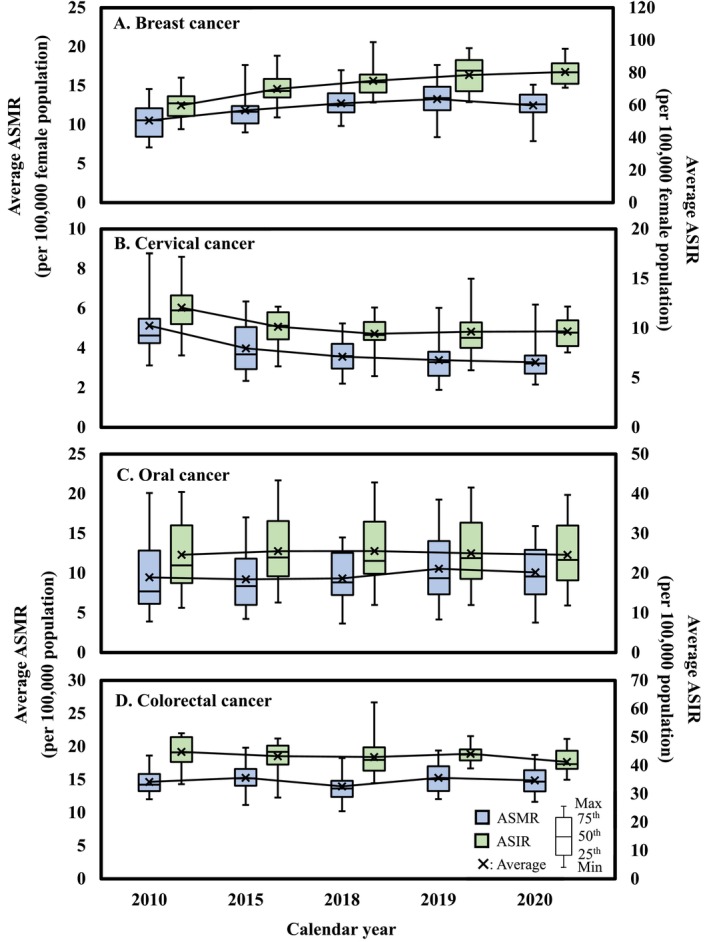
Annual trends in the average age‐standardized mortality rates (ASMR) and age‐standardized incidence rates (ASIR) for (A) breast cancer, (B) cervical cancer, (C) oral cancer, and (D) colorectal cancer.

The annual trends and county scale analyses of four types of cancer in Taiwan are presented (Figure 2 and Table S5). The average ASMR for BC increased from 10.5 in 2010 to 13.3 in 2019, then decreased to 12.5 in 2020 (Figure 2A). In contrast, the annual trend for CxCa consistently declined from 5.1 in 2010 to 3.3 in 2020 (Figure 2B). For OC, the ASMR remained steady from 2010 (9.5) to 2018 (9.3), while a significant increase in 2019 (10.6) with a slight decline in 2020 (10.1) was found that was still higher than in 2010 (Figure 2C). CRC had the lowest ASMR at 14.0 in 2018, while it increased to 15.3 in 2019 and decreased to 14.9 in 2020 (Figure 2D).

For BC, Chiayi City, Taichung City, and Taipei City had the highest rates of 14.2, 13.8, and 13.2 per 100,000 female population, respectively. Taipei City led in the north, Taichung City in the central region, and Chiayi City in the south. For CxCa, Taitung County, Hualien County, and Hsinchu City had higher rates of 5.2, 4.8, and 4.4 per 100,000 female population, respectively. For OC, Taitung County, Hualien County, and Yunlin County had higher rates of 17.0, 15.4, and 14.5 per 100,000 population, respectively. Taitung and Hualien Counties led in the east, while Yunlin County had the highest rate in the central region. For CRC, Kaohsiung City (17.4) and Tainan City (17.3) in the south, and Chiayi City (16.7) in the south region had the highest rates.

The ASIR of oral, colorectal, breast, and CxCas for the years 2010, 2015, 2018, 2019, and 2020 also revealed (Figure 2). For BC, the ASIR increased from 59.9 in 2010 to 80.4 in 2020 (Figure 2A). For CxCa, the ASIR declined from 12.1 in 2010 to 9.4 in 2018, followed by a slight increase to 9.7 in 2020 (Figure 2B). The ASIR for OC showed a slight increase from 24.6 in 2010 to 25.6 in 2018, and then returned to 24.6 in 2020 (Figure 2C). For CRC, the ASIR decreased from 44.8 in 2010 to 41.3 in 2020 (Figure 2D).

For BC, the five‐year average ASIR was highest in Taipei City (91.2), New Taipei City (83.5), and Taichung City (80.1) per 100,000 female population. For CxCa, Pingtung County (12.4), Miaoli County (11.9), and Keelung City (11.8) had the highest five‐year average ASIR per 100,000 female population (Table S6). The ASIR for OC was highest in Taitung County (41.6), Hualien County (34.9), and Chiayi County (34.0) per 100,000 population. For CRC, Tainan City (49.5), Chiayi City (49.1), and Kaohsiung City (48.3) recorded the highest five‐year average ASIR per 100,000 population (Table S6).

### 3.2 Spatiotemporal Analysis for Cancer Burden

**FIGURE 3:**
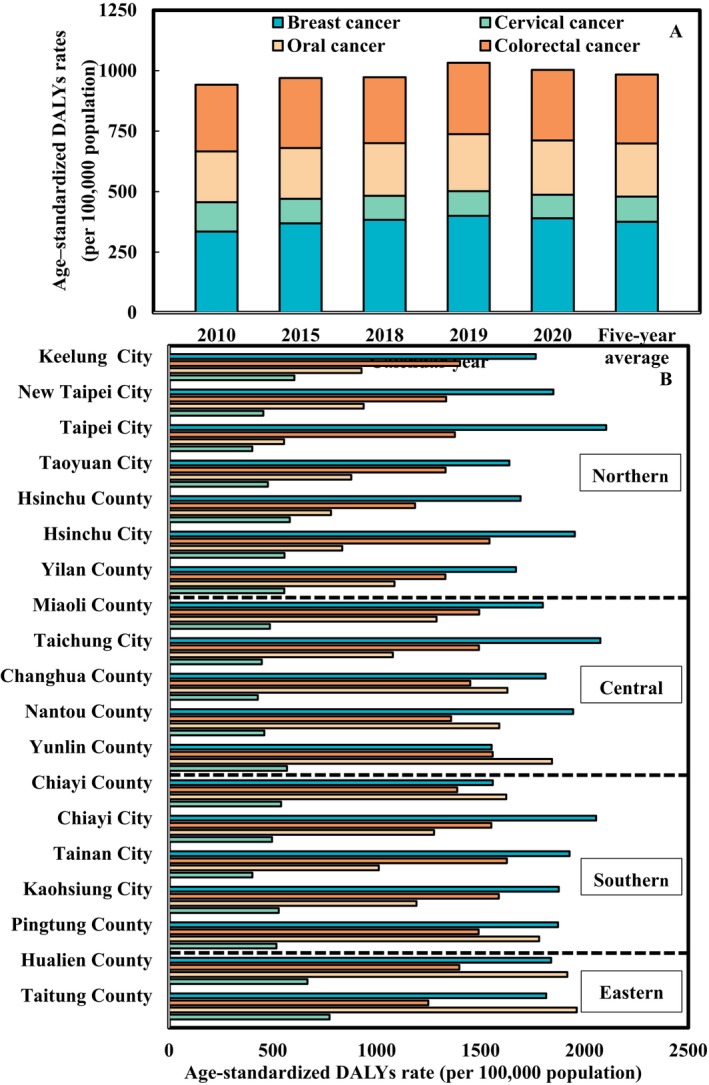
(A) Trends in age‐standardized disability‐adjusted life years (DALYs) rates for breast, oral, cervical, and colorectal cancers. (B) Five‐year cumulative age‐standardized disability‐adjusted life years (DALYs) rates for the four major cancers across various counties and cities in Taiwan.

Figure 3A provides a comprehensive overview of the trends in DALYs rates for BC, CxCa, OC, and CRC over the decade. The average DALYs rates during this period were 376 for BC, 104 for CxCa, 219 for OC, and 285 per 100,000 population for CRC. The data reveals an overall increase in DALYs rates for BC and CRC, a decline for CxCa, and slight fluctuations for OC. Specifically, the average contributions of each cancer type to the total DALYs burden from 2010 to 2020 are as follows: BC (38%), CRC (29%), OC (22%), and CxCa (11%) (Table S7).

**FIGURE 4:**
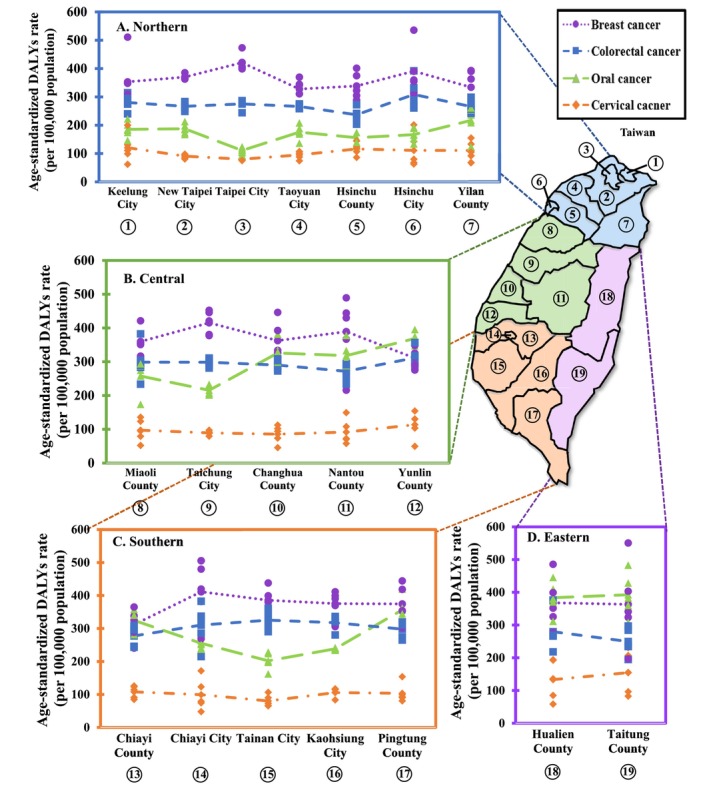
Age‐standardized DALYs rates for breast, cervical, oral, and colorectal cancers at the county level across Taiwan, grouped into four regions: (A) Northern, (B) Central, (C) Southern, and (D) Eastern Taiwan.

Figure 3B illustrates the county‐specific five‐year cumulative age‐standardized DALYs rates for the four major cancers. Overall, the cumulative burden of BC is particularly notable in Northern, Central, and Southern Taiwan. In contrast, OC shows a significant pattern in Eastern Taiwan (Taitung and Hualien Counties), as well as Yunlin and Chiayi Counties. Additionally, we present maps of the annual age‐standardized DALYs rates for BC, CxCa, OC, and CRC across Taiwan, categories by county and city into four regions: Northern, Central, Southern, and Eastern Taiwan (Figures 4,S2). Each mark in the figure represents a different calendar year, and the point on the line means the five‐year average. Our findings indicate that the highest burden of BC in 2019 was observed in Taitung County, with 551 DALYs. The Eastern Region, particularly Taitung County, also had the highest burden of OC. For CRC, the highest burden in 2019 was recorded in Hsinchu City, with 392 DALYs.

### 3.3 YLLs Contribution to DALYs

**FIGURE 5:**
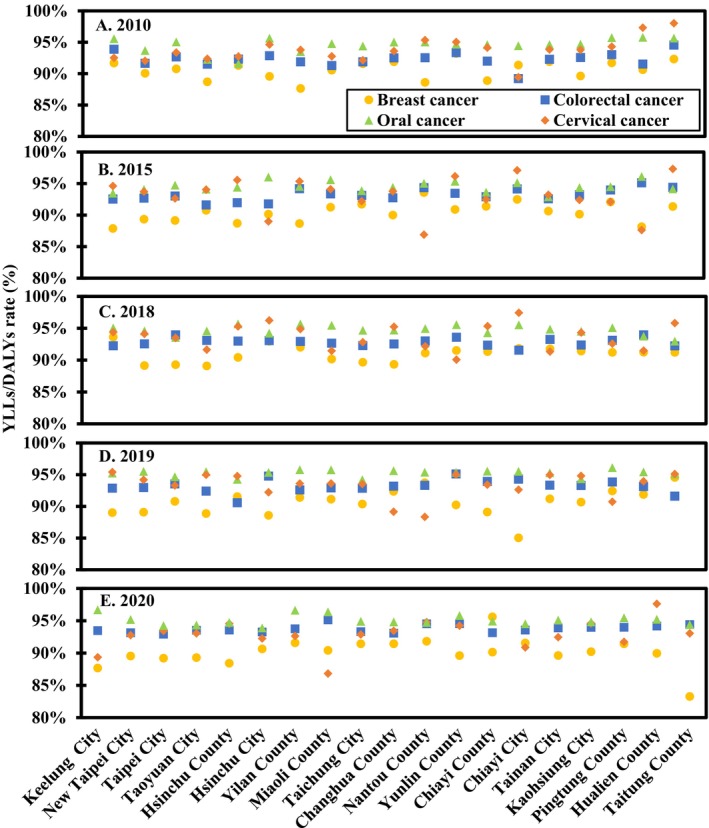
Percentage of years of life lost (YLLs) compared to age‐standardized disability‐adjusted life years (DALYs) rates for breast, oral, cervical, and colorectal cancers in various counties and cities across Taiwan for the years (A) 2010, (B) 2015, (C) 2018, (D) 2019, and (E) 2020.

Figure 5 shows the distribution of YLLs in age‐standardized DALYs rates for BC, CxCa, OC, and CRC across Taiwan counties for the years 2010, 2015, 2018, 2019, and 2020. For BC, the annual YLLs/DALYs ratio ranged from 90.1% to 91.1%. This high ratio means YLLs contributed significantly to DALYs, as the impact of death is weighted more heavily than disability. For CxCa, the ratio varied from 86.8% to 98.4%. A higher ratio indicates that a longer life expectancy at death results in a greater contribution of YLLs to DALYs. For OC, the ratio ranged from 94.6% to 95.3%, and for CRC, the ratio was highest in 2020, ranging from 92.3% to 93.8%. The reasons were the same as for BC and CxCa, highlighting the substantial impact of YLLs on the overall DALYs.

The ratio of YLLs/DALYs is an important indicator in burden of disease studies. This ratio helps to reveal the relative contributions of premature mortality and disability to the overall disease burden. A higher YLLs/DALYs ratio typically indicates that premature death is the major contributor to the burden, suggesting a need for interventions focused on reducing mortality, such as vaccination or early screening. On the other hand, a higher YLDs ratio highlights the long‐term impact of the disease on quality of life, emphasizing the need for strategies that focus on chronic disease management, rehabilitation, and improving quality of life.

### 3.4 Correlation Between Burden and Screening Rates

**FIGURE 6:**
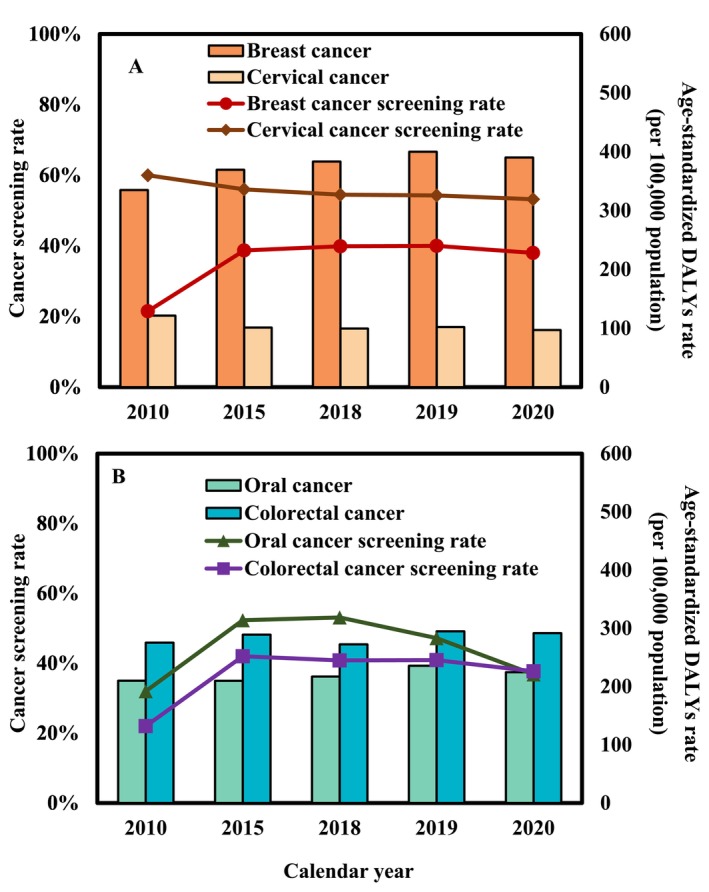
Age‐standardized DALY rates (per 100,000 population) alongside the annual screening rates in Taiwan for the years 2010, 2015, 2018, 2019, and 2020, for (A) breast and cervical cancers and (B) oral and colorectal cancers, respectively.

The age‐standardized DALYs rate with annual screening rates was shown in Figure 6. For BC, the average five‐year screening rate increased from 22% in 2010 to 38% in 2020, averaging 35.6%. This trend aligns with changes in the age‐standardized DALYs rate (Figure 6A). Conversely, for CxCa, the average 5‐year Pap smear screening rate was 55.6%, with a decrease from 60% in 2010 to 53% by 2020, yet maintaining a positive follow‐up completion rate of over 90%. This reduction in screening correlated with the most significant decline in disease burden among the four cancers between 2010 and 2020 (Figure 6A). The average screening rates for OC and CRC were 44% and 37%, respectively, while the screening rates in 2020 were likely to be decreased significantly due in part to COVID‐19 pandemic with stable and minimal changes in the age‐standardized DALYs rates (Figure 6B).

## 4 Discussion

The primary objective of this study was to analyze the burden of four major cancers of BC, CxCa, OC, and CRC in Taiwan from 2010 to 2020. Using the DALYs approach, we aimed to explore the temporal trends of age‐standardized mortality and incidence rates for these cancers. Additionally, we conducted a spatial analysis at the county level to examine regional disparities in cancer burden. By evaluating the contribution of YLLs to overall DALYs, we sought to identify key factors driving cancer‐related mortality. Lastly, we assessed the impact of screening programs on cancer burden and explored the relationship between screening rates and regional variations in disease outcomes across Taiwan.

The life expectancy data in our study were obtained from the national life table, which provides variable life expectancy based on birth year [18]. These life tables are typically derived from each country's demographic and health data, reflecting the actual life expectancy and health conditions of the local population. The advantage of this method is that the results accurately reflect the real‐life situation of the local population; however, it may underestimate potential gains in life expectancy. On the other hand, aspirational life tables assume ideal longevity, where individuals are expected to live in the best possible health conditions. This method emphasizes an idealized scenario of life expectancy, making it useful for cross‐country comparisons, but it may not align with the actual conditions of specific countries or regions. Recently, Oliveira et al. (2024) [29] conducted a systematic review and discussed the methodology of life tables. Among 18 studies, four studies performed their YLL calculations using national life tables [30, 31, 32, 33], while nine studies used aspirational life tables, representing the ideal standard [34, 35, 36]. Kassymbekova et al. (2024) [37] also noted that the YLL calculations based on life expectancy from the GBD study, which is significantly higher than that of Kazakhstan, might lead to an overestimation of YLL, DALYs, and the proportion of YLL to YLD. After carefully considering various YLL calculation methodologies, we chose to use the national life table for our analysis. This approach ensures that YLL estimates more accurately represent the actual health outcomes and life expectancy within the country.

Our results reveal that the burden in county scale analysis, which provides a more granular understanding of cancer burden variability across Taiwan. By analyzing the five‐year average age‐standardized DALYs rate and annual screening rates for BC, CxCa, OC, and CRC from 2018 to 2020 at the county scale (Figure S3), we identified significant disparities in both screening practices and cancer burden among counties. This detailed geographic analysis reveals that disparities in screening and DALYs rates can be linked to regional differences in socioeconomic status, healthcare access, education, occupation, and geographical location [38, 39, 40]. For example, our results show a positive correlation between the DALYs rate and screening rate for OC (*r* ^2^ = 0.20; *p* = 0.054; Figure S3), indicating that counties with higher OC screening rates tended to have consistent DALYs rates. This suggests that increased screening does not necessarily translate into lower DALYs rates, possibly due to other contributing factors such as delayed follow‐up or differences in cancer stage at detection. In contrast, for CxCa, a negative correlation was observed between the DALYs rate and screening rate (*r* ^2^ = 0.27; *p* = 0.022; Figure S3), reflecting the effectiveness of long‐standing screening policies that have led to earlier detection and treatment, thereby reducing the disease burden as seen in the declining age‐standardized mortality and incidence rates for CxCa. These insights are critical for policymakers to develop more localized strategies that can improve cancer outcomes by enhancing screening participation and addressing the barriers to early detection and treatment across different counties.

Another important point of discussion is the various factors that impact screening rates. Screening rates varied across counties due to complex factors, including socioeconomic status [38], healthcare access [39], education, occupation, and geographical location [40]. Moreover, the study also reveals the impact of the initial COVID‐19 pandemic in 2020 on cancer burden. Previous studies have examined the effects of the COVID‐19 pandemic on cancer screening numbers in Taiwan [41, 42]. These studies reported a significant 70% decrease in overall cancer screenings from 2019 to 2021, with specific reductions of 65% for CRC, 83% for OC, 70% for CxCa, and 76% for BC [41]. The decline in screening was observed across all hospital levels but was particularly pronounced in medical centers due to stricter policies, resulting in a more considerable reduction in screening rates [42].

However, this study has several limitations. First, there is no publicly available data on the number of BC, CxCa, OC, and CRC cases by stage and age group in Taiwan's 19 counties and cities. We assumed that the proportion of cancer stages by age group and county mirrors that of the entire country, which could lead to overestimation or underestimation of cases in different demographics. Second, the disability weights (*DW*) and duration (*L*_2_) used to calculate YLDs at each stage were sourced from international data, specifically the GBD 2019 Adolescent and Young Adult Cancer Collaborators (2022) [19] and Soerjomataram et al. (2012) [21]. Due to the lack of Taiwan‐specific parameters, these estimates may not fully capture local healthcare variations that could affect *DW* and *L*_2_. Lastly, the study did not include data on complications or post‐treatment sequelae affecting patients' quality of life, which could further impact the disease burden.

Our contribution was to quantitatively explore the major cancer burdens at the county scale and to detect its potential impacts based on DALYs assessment. In the context of the existing county‐specific data, we provide an example and methodology guidance for the comprehensive and systematic accounting of DAYLs‐based cancer burden perspectives at the county scale. Our results have potential implications to the cancer burden analysis at other scales and in other regions. Future work should be conducted to study the spatial–temporal pattern and driving factors of the cancer burdens and to predict future scenarios based on DALYs assessment model at the county scale.

## 5 Conclusions

This study provides a comprehensive assessment of the burden of breast, cervical, oral, and CRCs in Taiwan at the county scale using DALYs as a measure. The increasing DALYs for BC and CRC, alongside the high burden of OC in eastern counties, highlight the need for targeted public health strategies and resource distribution to reduce these disparities. The decrease in CxCa burden suggests the positive impact of national screening programs but underscores the need for sustained efforts. The data also reflect the adverse impact of the COVID‐19 pandemic on cancer control efforts, as seen in reduced screening rates and a corresponding increase in DALYs. Future efforts should focus on enhancing screening accessibility, particularly in high‐burden counties, and addressing socioeconomic and risk factors contributing to these disparities. This study provides valuable insights for policymakers and public health officials to develop region‐specific cancer prevention and control strategies.

## Author Contributions

**Cheng‐Chieh Hsieh:** conceptualization (equal), data curation (equal), methodology (equal), visualization (equal), writing – original draft (supporting), writing – review and editing (equal). **Si‐Yu Chen:** methodology (equal), validation (supporting), writing – original draft (equal), writing – review and editing (equal). **Chun‐Hui Lin:** validation (supporting), writing – original draft (equal), writing – review and editing (equal). **Szu‐Chieh Chen:** conceptualization (equal), data curation (equal), methodology (equal), visualization (lead), writing – original draft (lead), writing – review and editing (equal). **Chung‐Min Liao:** conceptualization (supporting), funding acquisition (equal), project administration (equal), supervision (equal), visualization (supporting), writing – review and editing (lead).

## Conflicts of Interest

The authors declare no conflicts of interest.

## Supporting information


Appendix S1.

## Data Availability

The datasets used in this study are available from the corresponding author upon reasonable request.

## References

[1] WHO , Cancer Statistics (WHO, 2022), https://www.who.int/news‐room/fact‐sheets/detail/cancer.

[2] International Agency for Research on Cancer , “Global Cancer Observatory: Cancer Today (Version 1.1). International Agency for Research on Cancer,” 2024, https://gco.iarc.who.int/today.

[3] GBD 2021 Diseases and Injuries Collaborators , “Global Incidence, Prevalence, Years Lived With Disability (YLDs), Disability‐Adjusted Life‐Years (DALYs), and Healthy Life Expectancy (HALE) for 371 Diseases and Injuries in 204 Countries and Territories and 811 Subnational Locations, 1990–2021: A Systematic Analysis for the Global Burden of Disease Study 2021,” Lancet 403, no. 10440 (2024): 2133–2161, 10.1016/S0140-6736(24)00757-8. 38642570 PMC11122111

[4] C. J. Murray , “Quantifying the Burden of Disease: The Technical Basis for Disability‐Adjusted Life Years,” Bulletin of the World Health Organization 72, no. 3 (1994): 429–445. 8062401 PMC2486718

[5] S. Xu , Y. Liu , T. Zhang , et al., “The Global, Regional, and National Burden and Trends of Breast Cancer From 1990 to 2019: Results From the Global Burden of Disease Study 2019,” Frontiers in Oncology 11 (2021): 689562, 10.3389/fonc.2021.689562. 34094989 PMC8176863

[6] R. Sun , W. Dou , W. Liu , et al., “Global, Regional, and National Burden of Oral Cancer and Its Attributable Risk Factors From 1990 to 2019,” Cancer Medicine 12, no. 12 (2023): 13811–13820, 10.1002/cam4.6025. 37132165 PMC10315711

[7] N. V. S. Reis , B. B. Andrade , M. R. Guerra , M. T. B. Teixeira , D. C. Malta , and V. M. A. Passos , “The Global Burden of Disease Study Estimates of Brazil's Cervical Cancer Burden,” Annals of Global Health 86, no. 1 (2020): 56, 10.5334/aogh.2756. 32566484 PMC7292105

[8] Y. Yang , M. Zhou , X. Zeng , and C. Wang , “The Burden of Oral Cancer in China, 1990‐2017: An Analysis for the Global Burden of Disease, Injuries, and Risk Factors Study 2017,” BMC Oral Health 21, no. 1 (2021): 44, 10.1186/s12903-020-01386-y. 33509185 PMC7842005

[9] C. Bauer , K. Zhang , Q. Xiao , J. Lu , Y. R. Hong , and R. Suk , “County‐Level Social Vulnerability and Breast, Cervical, and Colorectal Cancer Screening Rates in the US, 2018,” JAMA Network Open 5, no. 9 (2022): e2233429, 10.1001/jamanetworkopen.2022.33429. 36166230 PMC9516325

[10] P. Charalampous , V. Gorasso , D. Plass , et al., “Burden of Non‐Communicable Disease Studies in Europe: A Systematic Review of Data Sources and Methodological Choices,” European Journal of Public Health 32, no. 2 (2022): 289–296, 10.1093/eurpub/ckab218. 35015851 PMC8975530

[11] W. C. Lo , T. H. Hu , C. Y. Shih , H. H. Lin , and J. S. Hwang , “Impact of Healthy Lifestyle Factors on Life Expectancy and Lifetime Health Care Expenditure: Nationwide Cohort Study,” JMIR Public Health and Surveillance 10 (2024): e57045, 10.2196/57045. 39018094 PMC11292159

[12] C. J. Chen , S. L. You , L. H. Lin , W. L. Hsu , and Y. W. Yang , “Cancer Epidemiology and Control in Taiwan: A Brief Review,” Japanese Journal of Clinical Oncology 32 (2002): S66–S81, 10.1093/jjco/hye138. 11959880

[13] C. J. Chiang , Y. C. Chen , C. J. Chen , S. L. You , M. S. Lai , and Taiwan Cancer Registry Task Force , “Cancer Trends in Taiwan,” Japanese Journal of Clinical Oncology 40, no. 10 (2010): 897–904, 10.1093/jjco/hyq057. 20495192

[14] Health Promotion Administration, Ministry of Health and Welfare , “Taiwan Breast Cancer, Oral Cancer, and Colorectal Cancer Screening Programs. Health Promotion Administration, Ministry of Health and Welfare,” 2021, https://www.hpa.gov.tw/EngPages/Detail.aspx?nodeid=1051&pid=5957.

[15] Health Promotion Administration, Ministry of Health and Welfare , “Cancer Prevention and Control in Taiwan. Health Promotion Administration, Ministry of Health and Welfare,” 2023, https://www.hpa.gov.tw/EngPages/Detail.aspx?nodeid=3840&pid=10530.

[16] Health Promotion Administration, Ministry of Health and Welfare , “Cancer Registry Annual Report. Health Promotion Administration, Ministry of Health and Welfare,” 2023, https://www.hpa.gov.tw/Pages/List.aspx?nodeid=119.

[17] Department of Statistics, Ministry of Health and Welfare , “Cause of Death Statistics. Department of Statistics, Ministry of Health and Welfare,” 2023, https://dep.mohw.gov.tw/dos/cp‐6602‐74526‐113.html.

[18] Ministry of the Interior, Taiwan , “Abridged Life Table in Republic of China Area. Ministry of the Interior, Taiwan,” 2021, https://www.moi.gov.tw/cl.aspx?n=3053.

[19] GBD 2019 Adolescent and Young Adult Cancer Collaborators , “The Global Burden of Adolescent and Young Adult Cancer in 2019: A Systematic Analysis for the Global Burden of Disease Study 2019,” Lancet Oncology 23, no. 1 (2022): 27–52, 10.1016/S1470-2045(21)00581-7. 34871551 PMC8716339

[20] Global Burden of Disease 2019 Cancer Collaboration , “Cancer Incidence, Mortality, Years of Life Lost, Years Lived With Disability, and Disability‐Adjusted Life Years for 29 Cancer Groups From 2010 to 2019: A Systematic Analysis for the Global Burden of Disease Study 2019,” JAMA Oncology 8, no. 3 (2022): 420–444, 10.1001/jamaoncol.2021.6987. 34967848 PMC8719276

[21] I. Soerjomataram , J. Lortet‐Tieulent , J. Ferlay , et al., “Estimating and Validating Disability–Adjusted Life Years at the Global Level: A Methodological Framework for Cancer,” BMC Medical Research Methodology 12 (2012): 125, 10.1186/1471-2288-12-125. 22901001 PMC3490831

[22] V. L. Allgar and R. D. Neal , “Delays in the Diagnosis of Six Cancers: Analysis of Data From the National Survey of NHS Patients: Cancer,” British Journal of Cancer 92, no. 11 (2005): 1959–1970, 10.1038/sj.bjc.6602587. 15870714 PMC2361797

[23] Surveillance, Epidemiology, and End Results (SEER) Program , “SEER*Stat Database: Incidence—SEER 18 Regs Research Data + Hurricane Katrina Impacted Louisiana Cases, Nov 2012 Sub (1973–2010 Varying)—Linked to County Attributes—Total U.S., 1969–2011 Counties, National Cancer Institute, DCCPS, Surveillance Research Program, Surveillance Systems Branch, Released April 2013, Based on the November 2012 Submission,” 2021, www.seer.cancer.gov.

[24] R. D. Neal , N. U. Din , W. Hamilton , et al., “Comparison of Cancer Diagnostic Intervals Before and After Implementation of NICE Guidelines: Analysis of Data From the UK General Practice Research Database,” British Journal of Cancer 110, no. 3 (2014): 584–592, 10.1038/bjc.2013.791. 24366304 PMC3915139

[25] Ministry of Health and Welfare , “Taiwan Health and Welfare Report. Ministry of Health and Welfare,” 2022, https://www.mohw.gov.tw/lp‐3196‐1.html.

[26] Ministry of the Interior, Taiwan , “Population by Age for Counties and Cities. Ministry of the Interior, Taiwan,” 2024, https://www.ris.gov.tw/app/portal/346.

[27] WHO , “Age Standardization of Rates: A New WHO Standard,” 2001, https://seer.cancer.gov/stdpopulations/world.who.html.

[28] B. Devleesschauwer , P. Charalampous , V. Gorasso , et al., “Standardised Reporting of Burden of Disease Studies: The STROBOD Statement,” Population Health Metrics 22, no. 1 (2024): 28, 10.1186/s12963-024-00347-9. 39375690 PMC11459887

[29] C. C. Oliveira , P. Charalampous , J. Delaye , et al., “A Systematic Review of Studies That Estimated the Burden of Chronic Non‐Communicable Rare Diseases Using Disability‐Adjusted Life Years,” Orphanet Journal of Rare Diseases 19, no. 1 (2024): 333, 10.1186/s13023-024-03342-3. 39252105 PMC11384705

[30] H. Abolhassani , A. Aghamohammadi , F. Abolhassani , et al., “Health Policy for Common Variable Immunodeficiency: Burden of the Disease,” Journal of Investigational Allergology & Clinical Immunology 21, no. 6 (2011): 454–458. 21995178

[31] M. Inês , T. Coelho , I. Conceição , F. Landeiro , M. de Carvalho , and J. Costa , “Societal Costs and Burden of Hereditary Transthyretin Amyloidosis Polyneuropathy,” Amyloid 27, no. 2 (2020): 89–96, 10.1080/13506129.2019.1701429. 31854198

[32] C. Janphram , S. Worawichawong , S. Boongird , U. Udomsubpayakul , M. Assanatham , and C. Kitiyakara , “Year of Life Lost due to Premature Death From Glomerulonephritis in Thailand,” Journal of the American Society of Nephrology 32 (2021): 491–492, 10.1681/ASN.20213210S1491d.

[33] K. Kansal , M. Mareddy , K. Sloane , et al., “Survival in Frontotemporal Dementia Phenotypes: A Meta‐Analysis,” Dementia and Geriatric Cognitive Disorders 41 (2016): 109–122, 10.1159/000443205. 26854827

[34] GBD 2019 Diseases and Injuries Collaborators , “Global Burden of 369 Diseases and Injuries in 204 Countries and Territories, 1990–2019: A Systematic Analysis for the Global Burden of Disease Study 2019,” Lancet 396, no. 10258 (2020): 1204–1222, 10.1016/S0140-6736(20)32226-1. 33069326 PMC7567026

[35] A. Café , M. Carvalho , M. Crato , et al., “Haemophilia A: Health and Economic Burden of a Rare Disease in Portugal,” Orphanet Journal of Rare Diseases 14, no. 1 (2019): 211, 10.1186/s13023-019-1175-5. 31484564 PMC6727364

[36] GBD 2016 Motor Neuron Disease Collaborators , “Global, Regional, and National Burden of Motor Neuron Diseases 1990–2016: A Systematic Analysis for the Global Burden of Disease Study 2016,” Lancet Neurology 17, no. 12 (2018): 1083–1097, 10.1016/S1474-4422(18)30404-6. 30409709 PMC6234315

[37] F. Kassymbekova , N. Glushkova , G. Dunenova , et al., “Burden of Major Cancer Types in Almaty, Kazakhstan,” Scientific Reports 14, no. 1 (2024): 20536, 10.1038/s41598-024-71449-5. 39232186 PMC11375099

[38] W. Y. Lin , P. Y. Lin , W. M. Liang , and H. W. Kuo , “Relative and Absolute Inequalities in Cerebrovascular Disease Mortality Rates: Exploring the Influence of Socioeconomic Status and Urbanization Levels in Taiwan,” BMC Public Health 24 (2024): 1186, 10.1186/s12889-024-18679-4. 38678225 PMC11055299

[39] T. Y. Lin and H. W. Yu , “Spatial Analysis of Home and Community‐Based Services and Number of Deaths Among Older Adults in Taiwan,” Journal of Applied Gerontology 43, no. 3 (2023): 261–275, 10.1177/07334648231214911. 38086745

[40] L. Song and N. Lin , “Social Capital and Health Inequality: Evidence From Taiwan,” Journal of Health and Social Behavior 50, no. 2 (2009): 149–163, 10.1177/002214650905000203. 19537457

[41] C. H. Su , P. S. Hsu , and C. S. Lin , “Impact of the COVID‐19 Pandemic on Population‐Based Cancer Screening, a Nationwide Retrospective Study in Taiwan,” BMC Health Services Research 23, no. 1 (2023): 878, 10.1186/s12913-023-09901-x. 37605162 PMC10440859

[42] H. Y. Tsai , Y. L. Chang , C. T. Shen , W. S. Chung , H. J. Tsai , and F. M. Chen , “Effects of the COVID‐19 Pandemic on Breast Cancer Screening in Taiwan,” Breast 54 (2020): 52–55, 10.1016/j.breast.2020.08.014. 32919172 PMC7470863

