# Supplementary material for: Disability‐Adjusted Life Years (DALYs) due to Breast, Cervical, Colorectal and Oral Cancers in Taiwan Regions
Source: Cancer Med. 2025 Jan 7;14(1):e70592. doi: 10.1002/cam4.70592 (PMC11705416; doi:10.1002/cam4.70592)
Supplement: Supplementary file 1 — Appendix S1. [file CAM4-14-e70592-s001.docx]

**APPENDIX TABLE 1.** Cancer–specific disease weight (*D*) and disease duration (*L*) in four disease states (Diagnosis and treatment, Control, Pre–terminal, and terminal phases).

| **Disease weight in different disease states** | | | | | | | | | |
| --- | --- | --- | --- | --- | --- | --- | --- | --- | --- |
| **Cancer** | **Diagnosis & treatment *(D_D_)*** | | **Control**  ***(D_R_)*** | | | **Pre**–**terminal *(D_M_)*** | | **Terminal *(D_T_)*** | |
| Breast cancer | 0.288 ^a^  (0.193–0.399) ^b^ | | 0.049 ^a^  (0.031–0.072) ^b^ | | | 0.451 ^a^  (0.307–0.6) ^b^ | | 0.54 ^a^  (0.377–0.687) ^b^ | |
| Cervical cancer |  |  |  |  |  |  |  |  |  |
| Oral cancer |  |  |  |  |  |  |  |  |  |
| Colorectal cancer |  |  |  |  |  |  |  |  |  |
| **Disease duration (years)** | | | | | | | | | |
| **Cancer** | **Time to death *(T_D_)*** | **Time to cure (*T_C_)*** | | ***L_D_*** | ***L_R1_*^d^ / *L_R2_*^e^** | | ***L_M_*** | | ***L_T_*** |
| Breast cancer | 5.70 ^c^ | 7.00 ^c^ | | 0.25 | 6.75 / 3.89 | | 1.475 | | 0.083 |
| Cervical cancer | 10.00 | 10.00 | | 0.40 | 9.60 / 8.75 | | 0.767 | | 0.083 |
| Oral cancer | 3.00 ^c^ | 7.00 ^c^ | | 0.44 | 6.56 / 2.09 | | 0.775 | | 0.083 |
| Colorectal cancer | 1.60 ^c^ | 7.00 ^c^ | | 0.33 | 6.67 / 0.375 | | 0.808 | | 0.083 |

**^a^** Mean value^1^

**^b^** 95% uncertainty interval (UI)^1^

**^c^** Adopted from Soerjomataram et al. (2012)^2^.

**^d^** $L_{R1}=T_{C}-L_{D}$


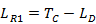


**^e^** $L_{R2}=T_{D}-L_{(D.M.T)}$


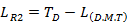


**APPENDIX TABLE 2.** 2010–2020 average proportion treated (*P*, %) at different cancer stage (stage 0, I, II, III, and IV) and age groups for oral cancer, colorectal cancer, breast cancer, and cervical cancer.

| Age groups | Stage 0 | Stage I | Stage II | Stage III | Stage IV |
| --- | --- | --- | --- | --- | --- |
| Oral cancer | | | | | |
| 0–39 yrs | 93 | 100 | 99 | 98 | 96 |
| 40–59 yrs | 98 | 99 | 99 | 98 | 96 |
| 60+ yrs | 95 | 99 | 98 | 96 | 93 |
| Average | 96 | 99 | 99 | 97 | 95 |
| Colorectal cancer | | | | | |
| 0–49 yrs | 100 | 99 | 100 | 99 | 93 |
| 50–69 yrs | 100 | 99 | 99 | 99 | 91 |
| 70+ yrs | 98 | 96 | 96 | 95 | 76 |
| Average | 99 | 98 | 98 | 98 | 87 |
| Breast cancer | | | | | |
| 0–34 yrs | 99 | 98 | 97 | 96 | 82 |
| 35–49 yrs | 99 | 99 | 98 | 96 | 76 |
| 50–69 yrs | 99 | 99 | 98 | 97 | 76 |
| 70+ yrs | 98 | 98 | 94 | 89 | 68 |
| Average | 99 | 99 | 97 | 95 | 76 |
| Cervical cancer | | | | | |
| 0–29 yrs | 98 | 98 | 96 | 100 | 100 |
| 30–49 yrs | 99 | 99 | 98 | 99 | 85 |
| 50–69 yrs | 99 | 99 | 99 | 97 | 91 |
| 70+ yrs | 97 | 98 | 97 | 95 | 80 |
| Average | 98 | 99 | 98 | 98 | 89 |

**APPENDIX TABLE 3.** 2010–2020 proportion cured (*S*, %) at different cancer stage (stage 0, I, II, III, and IV) and cancer sites.

| Cancer sites | Cancer stages | 2010 | 2015 | 2018 | 2019 | 2020 | Average (2010–2020) |
| --- | --- | --- | --- | --- | --- | --- | --- |
| Oral cancer | Stage 0 | 76.5 | 71.1 | 82.9 | 79.2 | 80.2 | 78.0 |
|  | Stage I | 77.8 | 80.2 | 85.1 | 85.0 | 85.3 | 82.7 |
|  | Stage II | 68.2 | 70.7 | 74.4 | 74.1 | 75.8 | 72.6 |
|  | Stage III | 51.4 | 55.0 | 60.6 | 61.1 | 61.1 | 57.8 |
|  | Stage IV | 32.8 | 33.8 | 37.7 | 37.3 | 37.7 | 35.9 |
| Colorectal cancer | Stage 0 | 85.4 | 86.5 | 95.1 | 94.6 | 94.6 | 91.2 |
|  | Stage I | 81.5 | 82.2 | 93.4 | 93.3 | 93.1 | 88.7 |
|  | Stage II | 71.8 | 70.7 | 84.6 | 83.3 | 83.9 | 78.9 |
|  | Stage III | 56.6 | 60.1 | 69.1 | 69.4 | 70.3 | 65.1 |
|  | Stage IV | 10.1 | 12.3 | 13.0 | 14.1 | 14.9 | 12.9 |
| Breast cancer | Stage 0 | 97.3 | 97.9 | 100.0 | 100.0 | 100.0 | 99.0 |
|  | Stage I | 95.5 | 96.0 | 100.0 | 100.0 | 100.0 | 98.3 |
|  | Stage II | 89.1 | 90.1 | 94.0 | 93.8 | 94.3 | 92.3 |
|  | Stage III | 70.6 | 73.9 | 77.9 | 78.5 | 79.5 | 76.1 |
|  | Stage IV | 25.2 | 28.1 | 34.1 | 34.8 | 34.9 | 31.4 |
| Cervical cancer | Stage 0 | 96.4 | 96.9 | 99.1 | 99.0 | 99.1 | 98.1 |
|  | Stage I | 84.0 | 88.2 | 88.5 | 89.5 | 90.0 | 88.0 |
|  | Stage II | 62.2 | 68.6 | 72.7 | 72.4 | 72.2 | 69.6 |
|  | Stage III | 39.0 | 56.5 | 61.5 | 60.5 | 61.0 | 55.7 |
|  | Stage IV | 17.6 | 19.7 | 24.3 | 23.1 | 22.3 | 21.4 |

# **APPENDIX TABLE 4.** Standardize reporting of burden of disease studies (STROBOD) checklist.

| **Item number** | **Domains and description of the recommended items** | **Reported on page number** |
| --- | --- | --- |
| Title | | |
| 1 | Identify the study as a burden of disease assessment by including keywords (e.g., Years of Life Lost, Years Lost due to Disability, Disability-Adjusted Life Years, burden of disease etc.), and describe the study setting | P1 |
| Abstract | | |
| 2 | Provide a summary of objectives, study setting, methods (including data sources and key methodological design choices used), results (including point estimates and, if applicable, uncertainty intervals), and conclusions | P2 |
| Introduction | | |
| 3 | Present background information to the study, its study aim(s), and its relevance for health policy or practice | P4 |
| Methods | | |
| Study setting | | |
| 4 | Report for which cause(s) the burden was calculated. Provide a case definition, e.g., in terms of an internationally recognized classification system such as the International Classification of Diseases and Related Health Problems 10th Revision | P5 |
| 5 | Report the reference population and any stratification of the reference population for the burden of disease assessment, i.e., the population for which the burden was calculated. This may include the geographical location (e.g., country or province/state), and whether the general population or a specific subset of the population (e.g., females, adolescents aged 10–19 years, etc.) was considered | P5 |
| 6 | Report the reference time period (e.g., year(s), month(s)) of the study. This refers to the time period to which the burden of disease estimates refer | P5 |
| Epidemiological and demographic input data | | |
| 7 | Report the sources, values, ranges, and, if used, probability distributions for all epidemiological input parameters. Report reasons or sources for distributions used to represent uncertainty where appropriate. Providing a (supplementary) table to show all epidemiological input parameters and respective sources and assumptions is strongly recommended | Appendix Table 2, Appendix Table 3 |
| 8 | Describe all possible data manipulations, such as bias corrections, data integration steps, or methods to ensure internal consistency of the data inputs | No |
| 9 | Report the sources and values of any population data used. If applicable, report the standard population used to calculate age-standardized rates | P9 |
| Daly methods | | |
| 10 | Report the age-conditional life expectancy used for calculating Years of Life Lost (i.e., national, regional, or aspirational life tables) or other methods (e.g., potential years of life lost, proportion of premature deaths under a selected age threshold etc.) | P6 |
| 11 | Report the perspective taken for calculating Years Lost due to Disability, i.e., incidence or prevalence perspective | Appendix Table 5, Appendix Table 6 |
| **Disease model** | | |
| 12 | Describe the disease model. Present and justify the included health outcomes and health states. Providing a (supplementary) figure visualizing the disease model is strongly recommended | Fig. 1, Appendix Fig. 1 |
| 13 | Report the source(s) and values of the used disability weights. Providing a (supplementary) table depicting the health states, brief lay descriptions, and the numerical values followed by its uncertainty intervals is strongly recommended | Appendix Table 1 |
| 14 | If new disability weights were elicited, provide information on how the health states were described and the elicitation procedures. As a minimum to the latter, describe which valuation technique was used and which reference group and size of the group (also known as panel of judges) evaluated the health states. Providing a supplementary table with a description of the valuation technique and brief lay descriptions used is strongly recommended | P7 |
| 15 | Report the source(s) and values of the used durations (if applicable). Providing a (supplementary) table depicting the health states and the numerical values followed by its uncertainty intervals is strongly recommended | Appendix Table 1 |
| 16 | Report the source(s) and values of the used conditional probabilities, severity distribution, and/or transition rates. Providing a (supplementary) table depicting the parent/child health outcomes and health states and the numerical values followed by its uncertainty intervals is strongly recommended | Appendix Table 1 |
| Multimorbidity adjustments | | |
| 17 | Report whether or not multimorbidity adjustments were applied to any of the input variables in the estimation of Years Lost due to Disability. If applied, describe which multimorbidity adjustment method was used | No |
| Social weighting factors | | |
| 18 | Report whether or not age weighting was applied. If applied, describe which parameters were used | No |
| 19 | Report whether or not time discounting was applied. If applied, describe which discount rate was used | No |
| Uncertainty and scenario analysis | | |
| 20 | Describe any methods used to perform uncertainty and variable importance (sensitivity) analyses. If, for example, Monte Carlo simulations were used, report the number of iterations | No |
| 21 | Describe any scenario analyses that were performed. Present the rationale and the alternative data inputs defining the alternative scenarios | No |
| Results | | |
| 22 | Report the point estimates and, if applicable, the uncertainty interval of the burden of disease estimates. Provide both absolute values, crude rates (optional), and age-standardized rates per 100,000 in a table or figure | Fig. 2  Appendix Table 5, Appendix Table 6 |
| 23 | If applicable, report the results of the scenario analyses. Tables and/or figures illustrating findings on the scenario analyses are strongly recommended | No |
| Discussion | | |
| 24 | Summarise the key study findings and describe how they support the conclusions reached | P15 |
| 25 | Discuss how the findings fit within current knowledge. Discuss potential implications for public health practice. Compare the results with those of other studies, and discuss methodological design differences, if relevant | P15 |
| 26 | Discuss strengths and limitations, and the generalisability of the study findings. If applicable, discuss the results of the uncertainty and scenario analyses | P15 |
| Open science | | |
| 27 | Make the source code or computational model(s) available as supporting information or via a dedicated open access repository (e.g., GitHub) | No |
| 28 | Describe how the study was funded and the role of the funder in the identification, design, conduct, and reporting of the analysis. Describe other non-monetary sources of support or any potential conflict(s) of interest of the study contributor(s) in accordance with the journal policy | P18 |

**APPENDIX TABLE 5.** The five–year average age–standardized mortality rates (ASMR) of breast, oral, cervical and colorectal cancers by county and city in Taiwan (Unit: per 100,000 population).

| County/City | Average ASMR for breast cancer | County/City | Average ASMR for cervical cancer | County/City | Average ASMR for oral cancer | County/City | Average ASMR for colorectal cancer |
| --- | --- | --- | --- | --- | --- | --- | --- |
| Chiayi City | 14.2 | Taitung County | 5.2 | Taitung County | 17.0 | Kaohsiung City | 17.4 |
| Taichung City | 13.8 | Hualien County | 4.8 | Hualien County | 15.4 | Tainan City | 17.3 |
| Taipei City | 13.2 | Hsinchu City | 4.4 | Yunlin County | 14.5 | Chiayi City | 16.7 |
| Pingtung County | 13.1 | Keelung City | 4.4 | Pingtung County | 13.7 | Yunlin County | 16.3 |
| Nantou County | 13.1 | Yilan County | 4.2 | Changhua County | 12.5 | Pingtung County | 16.0 |
| Hualien County | 12.9 | Pingtung County | 4.1 | Chiayi County | 12.3 | Hsinchu City | 15.8 |
| Tainan City | 12.8 | Hsinchu County | 4.1 | Nantou County | 12.1 | Taichung City | 15.5 |
| Kaohsiung City | 12.7 | Kaohsiung City | 4.0 | Chiayi City | 10.0 | Miaoli County | 15.5 |
| Keelung City | 12.6 | Yunlin County | 3.9 | Kaohsiung City | 9.1 | Changhua County | 14.9 |
| Taitung County | 12.5 | Miaoli County | 3.9 | Miaoli County | 9.0 | Chiayi County | 14.5 |
| Miaoli County | 12.2 | Chiayi City | 3.8 | Taichung City | 8.3 | Keelung City | 14.5 |
| New Taipei City | 12.0 | Chiayi County | 3.6 | Yilan County | 7.7 | Yilan County | 14.0 |
| Hsinchu City | 11.9 | Taichung City | 3.6 | Tainan City | 7.5 | Nantou County | 13.9 |
| Changhua County | 11.6 | Taoyuan City | 3.5 | New Taipei City | 6.9 | Hualien County | 13.6 |
| Hsinchu County | 11.1 | New Taipei City | 3.4 | Keelung City | 6.8 | Taoyuan City | 13.6 |
| Yilan County | 11.0 | Nantou County | 3.3 | Hsinchu City | 6.3 | New Taipei City | 13.4 |
| Taoyuan City | 10.5 | Changhua County | 3.3 | Taoyuan City | 6.2 | Taitung County | 13.2 |
| Yunlin County | 10.0 | Tainan City | 3.1 | Hsinchu County | 5.6 | Taipei City | 12.9 |
| Chiayi County | 10.0 | Taipei City | 2.7 | Taipei City | 3.9 | Hsinchu County | 12.5 |

**APPENDIX TABLE 6.** The five–year average age–standardized incidence rates (ASIR) of breast, oral, cervical and colorectal cancers by county and city in Taiwan (Unit: per 100,000 population).

| County/City | | Average ASIR for breast cancer | | County/City | | Average ASIR for cervical cancer | | County/City | Average ASMR for oral cancer | | County/City | | Average ASMR for colorectal cancer |
| --- | --- | --- | --- | --- | --- | --- | --- | --- | --- | --- | --- | --- | --- |
| Taipei City | 91.2 | | Pingtung County | | 12.4 | | Taitung County | | | 41.6 | | Tainan City | 49.5 |
| New Taipei City | 83.5 | | Miaoli County | | 11.9 | | Hualien County | | | 34.9 | | Chiayi City | 49.1 |
| Taichung City | 80.1 | | Keelung City | | 11.8 | | Chiayi County | | | 34.0 | | Kaohsiung City | 48.3 |
| Chiayi City | 79.5 | | Hsinchu City | | 11.2 | | Yunlin County | | | 33.4 | | Taichung City | 48.1 |
| Hsinchu City | 78.2 | | Nantou County | | 11.0 | | Changhua County | | | 32.2 | | Changhua County | 46.0 |
| Kaohsiung City | 76.5 | | Taichung City | | 10.5 | | Pingtung County | | | 31.9 | | Hsinchu City | 45.8 |
| Hualien County | 74.4 | | Hualien County | | 10.5 | | Nantou County | | | 30.9 | | Taoyuan City | 44.4 |
| Taoyuan City | 74.3 | | Hsinchu County | | 10.4 | | Kaohsiung City | | | 25.4 | | Miaoli County | 44.4 |
| Tainan City | 73.9 | | Yilan County | | 10.4 | | Chiayi City | | | 24.8 | | New Taipei City | 43.7 |
| Keelung City | 72.4 | | Taoyuan City | | 10.2 | | Taichung City | | | 23.5 | | Chiayi County | 43.3 |
| Miaoli County | 71.4 | | Kaohsiung City | | 10.2 | | Miaoli County | | | 21.8 | | Keelung City | 43.0 |
| Hsinchu County | 71.3 | | Chiayi County | | 9.9 | | Tainan City | | | 21.2 | | Pingtung County | 42.0 |
| Changhua County | 68.8 | | New Taipei City | | 9.7 | | Taoyuan City | | | 19.6 | | Taipei City | 41.1 |
| Yilan County | 67.2 | | Yunlin County | | 9.6 | | New Taipei City | | | 19.6 | | Yunlin County | 40.9 |
| Pingtung County | 65.8 | | Changhua County | | 9.0 | | Yilan County | | | 19.3 | | Yilan County | 40.4 |
| Taitung County | 65.2 | | Taitung County | | 8.8 | | Hsinchu County | | | 17.3 | | Hsinchu County | 39.9 |
| Nantou County | 65.0 | | Taipei City | | 8.7 | | Keelung City | | | 17.0 | | Nantou County | 38.5 |
| Chiayi County | 64.8 | | Chiayi City | | 8.6 | | Hsinchu City | | | 15.9 | | Hualien County | 38.4 |
| Yunlin County | 59.0 | | Tainan City | | 8.6 | | Taipei City | | | 11.9 | | Taitung County | 35.5 |

**APPENDIX TABLE 7.** The five–year average age–standardized DALYs rate of breast, oral, cervical and colorectal cancers in Taiwan (Unit: per 100,000 population).

| Year | Breast cancer | Cervical cancer | Oral cancer | Colorectal cancer |
| --- | --- | --- | --- | --- |
| 2010 | 335 | 122 | 210 | 275 |
| 2015 | 369 | 101 | 210 | 289 |
| 2018 | 383 | 100 | 217 | 272 |
| 2019 | 400 | 102 | 236 | 295 |
| 2020 | 390 | 97 | 225 | 292 |
| Five-year average (%)^a^ | 376 (38%) | 104 (11%) | 219 (22%) | 285 (29%) |

^a^ The percentage (%) represents the age-standardized DALYs rate of each cancer among the four types of cancers.

**APPENDIX TABLE 8.** The average service utilization rate, screening positive rate, and follow-up rates for positive case from 2018 to 2020^e,f^.

|  | Breast cancer (BC) | | | Cervical cancer (**CxCa)** | | | Oral cancer (OC) | | | Colorectal cancer (CRC) | | |
| --- | --- | --- | --- | --- | --- | --- | --- | --- | --- | --- | --- | --- |
| County/City | Screening rates (%) | Positive rate (%) | Follow-up rates (%) | Screening rates (%) | Positive rate (%) | Follow-up rates (%) | Screening rates (%) | Positive rate (%) | Follow-up rates (%) | Screening rates (%) | Positive rate (%) | Follow-up rates (%) |
| Taipei City ^a^ | 41.1 | 4.3 | 93.0 | 53.5 | 0.20 | 98.1 | 48.5 | 5.5 | 75.2 | 38.9 | 2.4 | 80.5 |
| Kaohsiung City ^d^ | 38.1 | 4.1 | 93.5 | 53.0 | 0.16 | 92.9 | 49.8 | 6.1 | 91.5 | 40.4 | 3.1 | 76.4 |
| Keelung City ^b^ | 43.3 | 5.4 | 93.8 | 54.9 | 0.20 | 97.7 | 30.0 | 6.6 | 80.4 | 42.2 | 3.1 | 76.0 |
| Hsinchu City | 45.3 | 3.9 | 92.5 | 53.9 | 0.22 | 92.8 | 32.0 | 8.9 | 81.5 | 41.7 | 2.6 | 78.0 |
| Taichung City ^a^ | 37.2 | 4.1 | 91.3 | 55.2 | 0.24 | 93.4 | 54.1 | 6.0 | 81.4 | 39.4 | 3.0 | 74.4 |
| Tainan City ^d^ | 40.9 | 4.5 | 92.3 | 54.8 | 0.20 | 94.5 | 53.1 | 6.1 | 80.3 | 41.8 | 3.3 | 75.4 |
| Chiayi City ^a^ | 41.2 | 3.8 | 93.4 | 53.2 | 0.19 | 92.4 | 54.1 | 9.5 | 76.6 | 45.2 | 3.2 | 79.9 |
| New Taipei City | 43.6 | 3.7 | 92.3 | 56.6 | 0.21 | 96.1 | 43.4 | 5.5 | 78.1 | 39.2 | 2.9 | 76.9 |
| Taoyuan City | 41.0 | 3.6 | 93.1 | 54.0 | 0.24 | 92.9 | 42.4 | 6.2 | 83.8 | 38.8 | 3.1 | 77.3 |
| Hsinchu County | 41.3 | 3.6 | 93.9 | 54.5 | 0.24 | 92.3 | 39.2 | 6.7 | 88.5 | 38.0 | 2.8 | 77.0 |
| Yilan County | 43.1 | 3.7 | 95.1 | 54.3 | 0.13 | 90.7 | 49.1 | 6.9 | 82.2 | 42.1 | 3.3 | 77.6 |
| Miaoli County | 32.6 | 3.9 | 93.3 | 49.9 | 0.29 | 81.9 | 34.2 | 7.1 | 82.4 | 33.3 | 3.2 | 70.7 |
| Changhua County | 39.4 | 3.5 | 91.2 | 57.0 | 0.21 | 91.3 | 62.9 | 7.1 | 84.3 | 42.7 | 3.0 | 75.8 |
| Nantou County | 35.8 | 4.9 | 91.8 | 49.3 | 0.19 | 90.6 | 41.6 | 7.7 | 87.1 | 37.0 | 3.0 | 69.4 |
| Yunlin County ^c, d^ | 28.2 | 4.8 | 89.0 | 49.1 | 0.25 | 85.3 | 39.0 | 9.1 | 75.3 | 36.2 | 3.3 | 69.4 |
| Chiayi County | 39.5 | 2.8 | 91.7 | 52.1 | 0.21 | 91.9 | 49.3 | 7.9 | 80.1 | 41.1 | 3.5 | 76.7 |
| Pingtung County | 34.1 | 4.0 | 93.1 | 49.7 | 0.22 | 92.0 | 55.9 | 9.0 | 85.0 | 38.9 | 3.5 | 76.0 |
| Hualien County ^b, c^ | 34.2 | 4.7 | 88.3 | 51.0 | 0.18 | 89.2 | 73.6 | 6.4 | 80.3 | 41.3 | 3.1 | 65.2 |
| Taitung County ^b, c^ | 35.3 | 3.6 | 92.0 | 47.2 | 0.19 | 89.9 | 48.2 | 7.6 | 83.5 | 37.8 | 3.7 | 72.0 |
| Overall | 39.6 | 4.0 | 92.5 | 54.0 | 0.21 | 93.4 | 50.2 | 6.8 | 81.9 | 39.5 | 3.0 | 75.7 |

^a, b, c, d^ Counties and cities with a high burden of breast, cervical, oral, and colorectal cancers in this study, respectively.

^e^ Screening rates, positive rate, and follow-up rates were assessed for women aged 45-69 who received mammography within the past two years, for women aged 30-69 who underwent cervical smear screening, for oral mucosal examinations among women aged 30-69, and for colorectal cancer screening among women aged 50-69 within the past two years. An underline indicates that the estimated value is higher than the overall value.

^f^ Adopted from Health Promotion Agency, Statistics of Health Promotion 2021.^3^

**APPENDIX FIGURE 1**

**Cured (included Sequelae)**

**Incidence**

**Death**

**Time to cured (*T_C_* )**

**Diagnosis & Treatment**

**(*L_D,_ D_D_*)**

**Remission**

**(*L_R1,_ D_R_*)**

**Remission**

***(L_R2 ,_ D_R_*)**

**Metastasis /Pre–terminal**

**(*L_M_*_,_ *D_M_*)**

**Terminal**

***(L_T_*, *D_T_* )**

*proportion*

*treated* ***(P)***

***1-P***

*proportion*

*cured* **(*S)***

***1-S***

**Time to death (*T_D_* )**

**APPENDIX FIGURE 2**

**
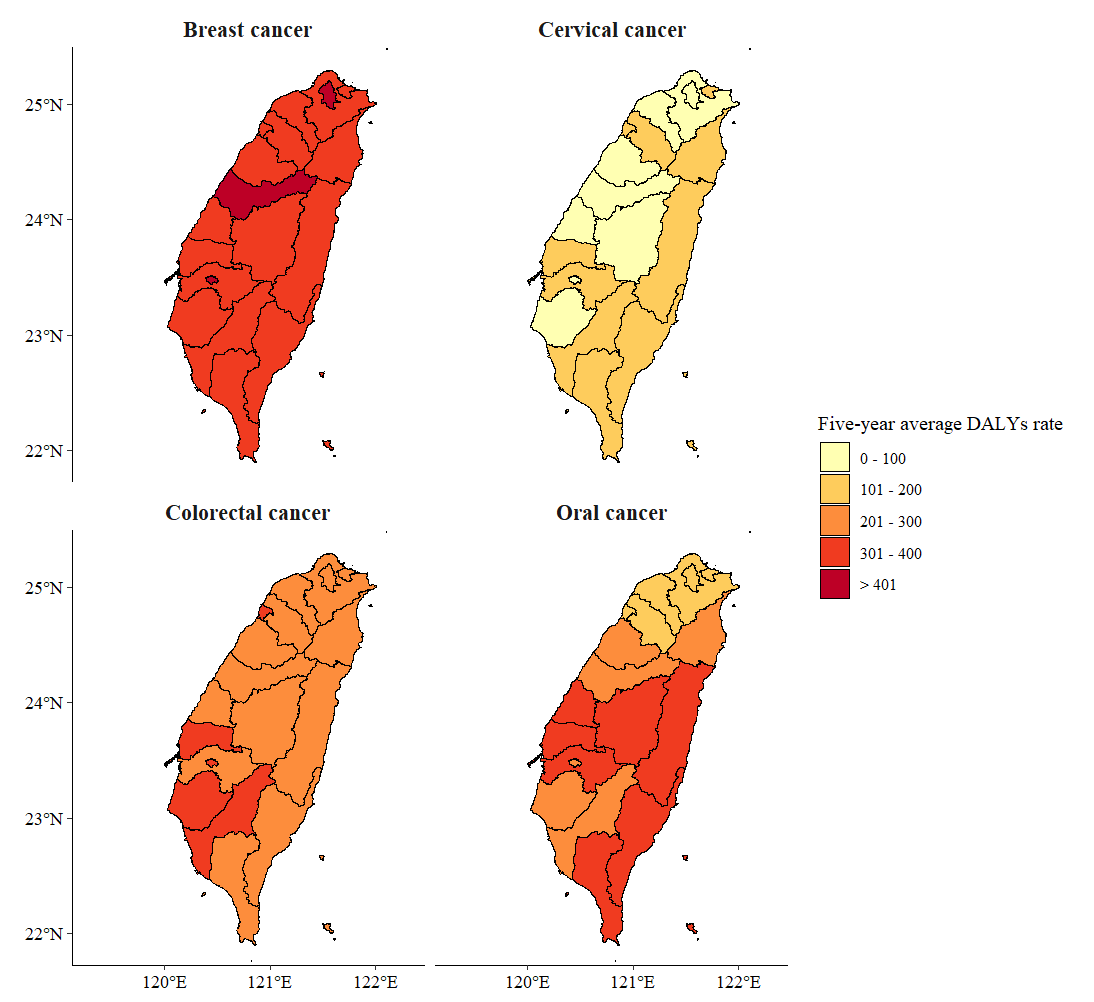
**

**APPENDIX FIGURE 3**

**REFERENCES**

1. GBD 2019 Adolescent and Young Adult Cancer Collaborators. The global burden of adolescent and young adult cancer in 2019: a systematic analysis for the Global Burden of Disease Study 2019. *Lancet Oncol.* 2022;23(1):27–52. doi:10.1016/S1470-2045(21)00581-7
2. Soerjomataram I, Lortet–Tieulent J, Ferlay J, et al. Estimating and validating disability–adjusted life years at the global level: a methodological framework for cancer. *BMC Med Res Methodol.* 2012;12:125. doi:10.1186/1471-2288-12-125
3. Health Promotion Agency, Statistics of Health Promotion 2021. Accessed Aug 31, 2024. <https://reurl.cc/VzRzg6>.
